# Engineering a HER2-CAR-NK Cell Secreting Soluble Programmed Cell Death Protein with Superior Antitumor Efficacy

**DOI:** 10.3390/ijms24076843

**Published:** 2023-04-06

**Authors:** Wenjiao Xia, Jiaxin Chen, Wenqing Hou, Junsheng Chen, Ying Xiong, Hongyan Li, Xin Qi, Hui Xu, Zuoquan Xie, Mingfeng Li, Xiaomin Zhang, Jing Li

**Affiliations:** 1 Key Laboratory of Marine Drugs, Chinese Ministry of Education, School of Medicine and Pharmacy, Ocean University of China, Laboratory for Marine Drugs and Bioproducts of Qingdao National Laboratory for Marine Science and Technology, Qingdao 266003, China; 2 School of Pharmacy, Fudan University, Shanghai, 201203, China; 3 State Key Laboratory of Drug Research, Shanghai Institute of Materia Medica, Chinese Academy of Sciences, 555 Zu Chong Zhi Road, Shanghai, 200126, China

**Keywords:** breast cancers, cytotherapy, chimeric antigen receptor, NK cells, sPD-1

## Abstract

A new therapy strategy for relapsing patients who have received trastuzumab treatment urgently needs to be explored. HER2-specific chimeric antigen receptor (CAR)-expressing NK cells are being rapidly developed for solid tumor therapy, as they have many advantages over HER2-CAR-T cells. Endogenous soluble PD-1 (sPD-1) from the PD-1 extracellular domain blocks PD-1/PD-L1 interaction to promote cancer immunology. Herein, we engineered a new HER2-CAR-NK cell that co-expresses sPD-1 (designed as sPD-1-CAR-NK cells) and assessed its cytotoxic activities toward various cancer cells, activation of immunity and sPD-1 release in vitro and in mouse models bearing breast cancer cells with high HER2 expression, with or without trastuzumab resistance. We demonstrated that sPD-1-CAR-NK cells were able to release bioactive sPD-1, thereby enhancing the cytolytic activities of HER2-CAR-NK cells against HER2 and PD-L1 highly expressing target cells accompanied by increases in the secretion of perforin, granzyme B and IFN-γ. In vivo, sPD-1-CAR-NK cells had superior immunological anticancer efficacy compared to HER2-CAR-NK cells, and they had advantages over HER2-CAR-NK cells in the intraperitoneal injection of sPD-1. Moreover, the infiltration and activation of NK and T cells into tumor tissue were increased in mice with sPD-1-CAR-NK cells. There was no significant change in the body temperature, organ tissue and body weight in all groups except for the group with the PD-1 injection. Together, these data indicate that HER2-specific sPD-1-CAR-NK cells can transport sPD-1 into cancer tissues with high HER2 expression, further improving the efficacy of HER-CAR-NK cells without obvious side effects. sPD-1-CAR-NK is a promising cytotherapeutic agent for patients bearing HER2-positive breast cancer, including those with trastuzumab resistance.

## 1. Introduction

Human epidermal growth factor receptor 2 (HER2)-positive breast cancer, due to the overexpression and/or amplification of the HER2-neu gene, accounts for around 20% of all breast cancers and has a poor prognosis compared with other breast cancer subtypes [1]. Several classes of HER2-targeted agents have been developed for the treatment of HER2-positive (HER2+) breast cancer, including monoclonal antibodies (trastuzumab and pertuzumab), small-molecule tyrosine kinase inhibitors (lapatinib and neratinib) and antibody–drug conjugates (trastuzumab emtansine T-DM1) [2,3]. Although the outcomes of patients with HER2-positive diseases have improved dramatically, the disease will inevitably relapse because of acquired resistance to HER2-targeted therapies [4]. How to improve the therapeutic efficacy for relapsed patients remains an urgent problem to be solved. 

The cell-membrane-bound molecule programmed death protein 1 (PD-1), expressed on T cells, natural killer (NK) cells, monocytes, dendritic cells (DCs), B cells and Tregs, has been found to interact with its ligand PD-L1 to mediate negative regulatory signals that effectively impede antitumor immune responses [5,6]. PD-L1 is highly expressed in most cancer cells and is upregulated by some cytokines in the tumor microenvironment, such as IFN-γ from activated immune cells [7]. Anti-PD-1 therapy has been found to reverse T lymphocyte depletion and restore antitumor immunity [8]. In addition to forming transmembrane proteins such as PD-1, soluble PD-1 detected in the plasma is produced either by the selective splicing of genes encoding the membrane-bound molecules or by the proteolytic cleavage of membrane-bound proteins [9]. sPD-1 has been found to function as a blocker of PD-1 ligands and suppress the interactions of PD-1 with PD-L1 and PD-L2 [10].

Many studies have shown that the immunosuppressive signal of PD-1 impairs the antitumor function of CAR-T cells [11]. CAR-T cells secreting single-chain variable fragments (scFv) of PD-1 antibodies enhance T cell expansion and effector function both in vitro and in vivo, bringing a new perspective to the CAR T cells’ strategy by combining with the checkpoint blockade [12,13]. Compared with monoclonal antibodies, soluble PD-1, the PD-1 extracellular domain, is an endogenous molecule, which would not induce immunological rejection in humans [14,15].

CAR-NK therapy has gained great attention owing to the lack of adverse effects observed in CAR-T therapies and to NK cells’ unique mechanisms in recognizing target cells [16,17]; furthermore, CAR-NK can be made into an off-the-shelf product for many recipients [18]. Immunotherapy using HER2-specific chimeric antigen receptor (CAR)-expressing NK cells has recently been rapidly developed for solid tumor therapy with significant antitumor effects in preclinical reports [19,20]. However, a direct involvement of sPD-1 in unleashing PD-1 immunosuppression by HER2-specific CAR-NK cells has not yet been demonstrated [21]. 

Herein, we engineered a new HER2-specific CAR composed of a PD-1 extracellular domain (sPD-1-CAR-NK cells) with the aim to improve the anticancer efficacy of HER2-CAR-NK cells for patients harboring cancer cells with high HER2 expression, especially for those with trastuzumab resistance. By investigating the cytotoxic activities, immune activation and sPD-1 release of sPD-1-CAR-NK cells in vitro and in vivo, it was found that sPD-1-CAR-NK cells had more potential immunological anticancer efficacy than HER2-CAR-NK cells, and advantages over HER2-CAR-NK cells, with the addition of sPD-1 in both immunocompetent mouse models and humanized mouse models.

## 2. Results

### 2.1. Generation of HER2-CAR-NK Cells and sPD-1-CAR-NK Cells

**Figure 1:**
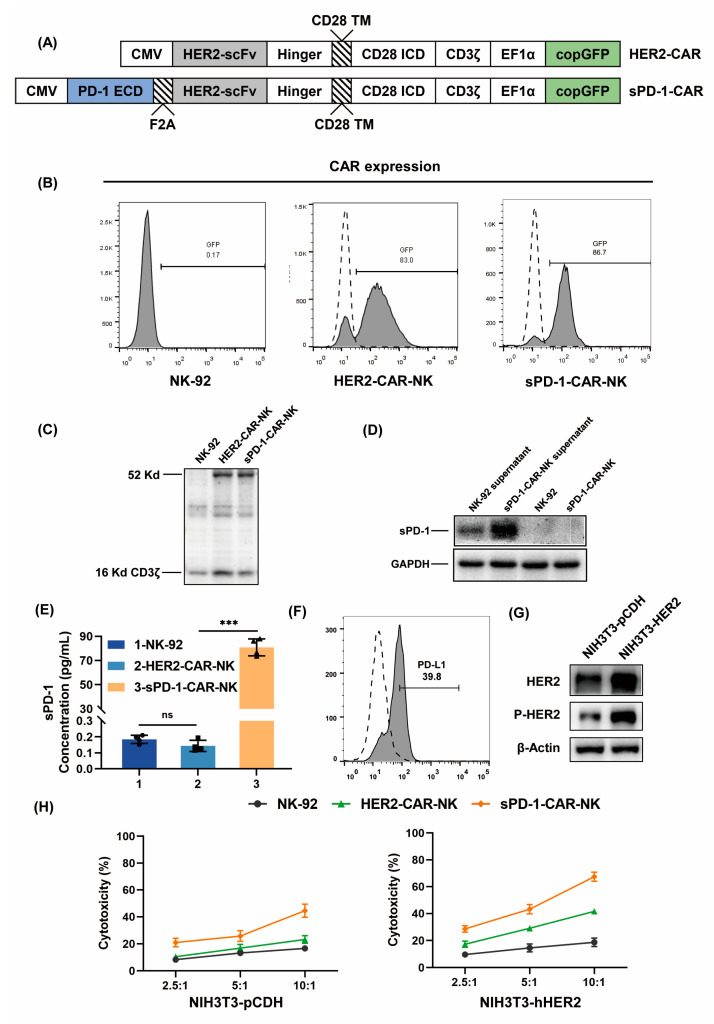
Generation of HER2-CAR-NK cells and sPD-1-CAR-NK cells. (**A**) Schematic representation of the lentiviral cassette encoding the HER2-CAR and sPD-1-CAR. PD1 ECD, an extracellular domain fragment of PD-1; F2A, 2A self-cleaving peptides; HER2 scFv, a single-chain variable fragment targeting HER2; Hinger, the hinge domain of the CD8α molecule; CD28 TM, the transmembrane region of the human CD28 molecule; CD28 ICD, the intracellular signaling domain of the human CD28 molecule; CD3ζ, the intracellular signaling domain of the CD3ζ molecule; copGFP, Cop Green fluorescent protein. CAR expression is driven by CMV promoter and EF1α promoter. (**B**) The expression of HER2-CAR and sPD-1-CAR on the surface of non-transduced (left) and transduced (middle and right) NK-92 cells were detected by flow cytometry. (**C**) Western blot analysis of the expression of CD3ζ in NK-92 cells, HER2-CAR-NK cells and sPD-1-CAR-NK cells. (**D**) Western blot analysis of the expression of sPD-1 in NK-92 cell supernatant, sPD-1-CAR-NK cell supernatant, NK-92 cells and sPD-1-CAR-NK cells. (**E**) NK-92 cells, HER2-CAR-NK cells and sPD-1-CAR-NK cells were cultured alone in αMEM medium containing 12.5% horse serum and 12.5% FBS for 24 h. The expression of sPD-1 was measured using an ELISA kit. (**F**) Flow cytometry of the expression of PD-L1 on the surface of NIH3T3-hHER2 cells. (**G**) Western blot analysis of the expression of HER2 and P-HER2 in NIH3T3-pCDH cells and NIH3T3-hHER2 cells. (**H**) NK-92 cells, HER2-CAR-NK cells and sPD-1-CAR-NK cells were placed in culture with NIH3T3-pCDH cells or NIH3T3-hHER2 cells at different effector–target ratios for 6 h. The cytotoxicity of NK-92 cells, HER2-CAR-NK cells and sPD-1-CAR-NK cells was measured by an LDH release assay. The data shown are representative of three experiments. Data represent the mean ± SD. n.s., nonsignificant; *** *p* < 0.001 by two-tailed Student’s *t*-test.

HER2-CAR-NK cells are stably transfected cells derived from parental NK-92 cells, constructed with a lentiviral vector encoding a second-generation CAR that contains HER2-specific single-chain antibodies scFv, the hinge domain of the CD8α molecule, a transmembrane region and intracellular signaling domain of the human CD28 molecule, and the intracellular signaling domain of the CD3ζ molecule. sPD-1-CAR was obtained by linking the sPD-1 extracellular domain (ECD, AA1-155) and 2A self-cleaving peptide (F2A) cassette to HER2-CAR (Figure 1A). After lentiviral transduction, the expression levels of the HER2-CAR and sPD1-CAR on parental NK-92 cells were obtained by detection of the co-expressed GFP, and we found that the transfection efficacy on NK-92 cells was in the range of 80–90% (Figure 1B). The CD3ζ chain is a 16-kDa transmembrane protein that is expressed primarily in NK and T cells, and is involved in the TCR–CD3 complex composition and the activation of lymphocytes [22]. This protein is required for the cell surface expression of the receptor [23]. The 16-kDa CD3ζ protein form representing endogenous non-phosphorylated CD3ζ was used as a loading control. Since both HER2-CAR and sPD-1-CAR contain CD3ζ sequences, together with other CAR elements, they form exogenous fusion proteins for expression. Thus, carrying the exogenous CD3ζ (52 kDa) protein can serve as a marker for HER2-CAR and sPD-1-CAR expression (Figure 1C). After 3 days of transfection, a very small amount of sPD-1 was detected in the culture supernatant of NK-92 cells and a large amount of sPD-1 was detected in the culture supernatant of sPD-1-CAR-NK cells, but no sPD-1 was detected in the lysate of either cell type (Figure 1D). Additionally, 80 pg/mL of sPD-1 was successfully detected in the culture supernatant of the sPD-1-CAR-NK cells (2 × 10^5^ cells) (Figure 1E). This suggests that sPD-1-CAR-NK cells can stably express sPD-1 and release it into the environment. To ensure that constructed sPD-1-CAR-NK cells specially and effectively targeted the HER2 antigen, and that secreted sPD-1 had an inhibitory effect on PD-L1 expressing cells, we transfected the vector of the human HER2 gene into mouse fibroblast NIH3T3 cells (Figure 1G), which express PD-L1 protein on the membrane [24]. Flow cytometric analysis confirms that the frequency of PD-L1 expression was 39.8% for NIH3T3-hHER2 (Figure 1F). NIH3T3 cells transfected with empty pCDH vector were used as controls for NIH3T3-hHER2 cells’ overexpression of HER2 (Figure 1G). As shown in Figure 1H, HER2-CAR-NK cells showed higher killing toxicity to NIH3T3-hHER2 cells than NIH3T3-pCDH cells with the same efficacy–target ratio, and sPD-1-CAR-NK cells were more effective than HER2-CAR-NK cells. These results indicate that the HER2-specific CAR-NK cells were established successfully, and they can express and release bioactive sPD-1 into the cellular environment.

### 2.2. sPD-1-CAR-NK Cells Enhance Cytotoxicity toward HER2- and PD-L1-Positive Breast Cancer Cells

**Figure 2:**
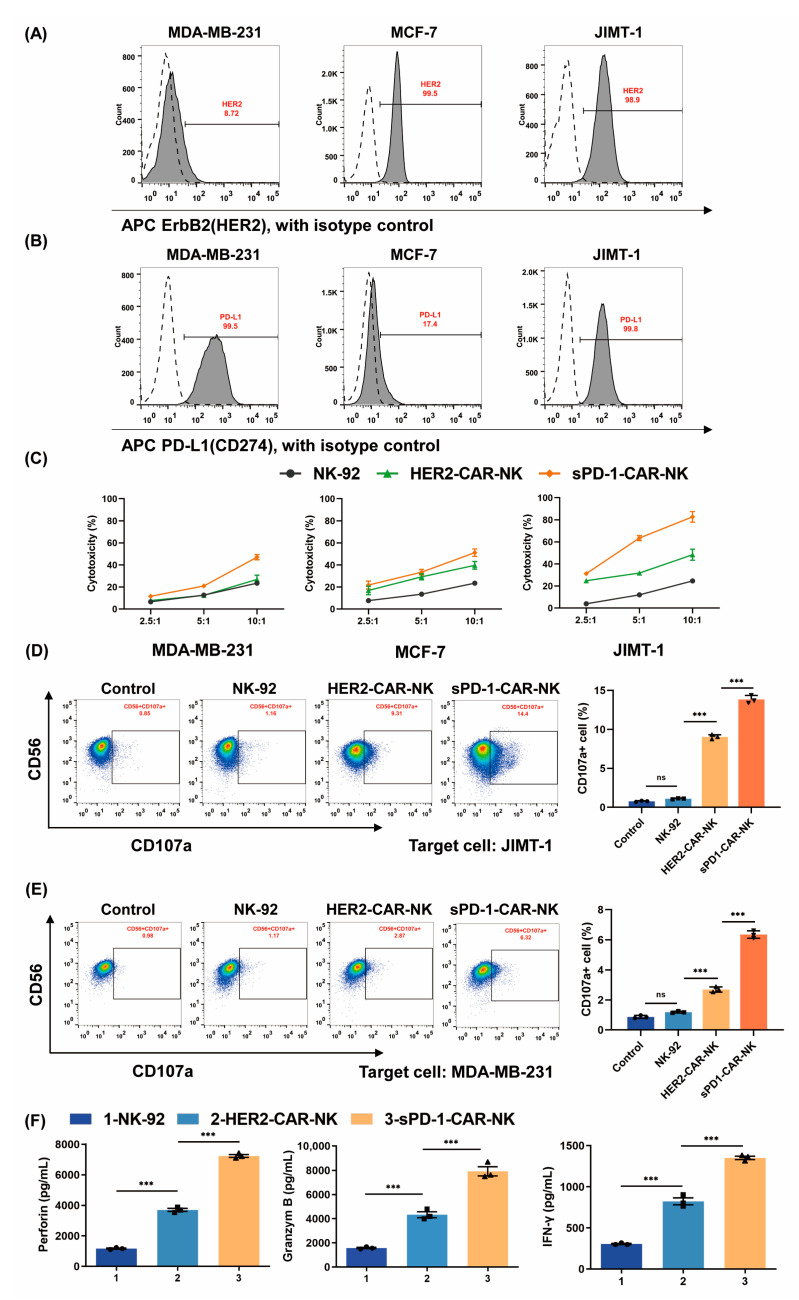
The cytotoxic capacity of sPD-1-CAR-NK cells is associated with the expression levels of HER2 and PD-L1 in cancer cells. (**A**,**B**) The expression of HER2 and PD-L1 on the surface of MDA-MB-231, MCF-7 and JIMT-1 cells. (**C**) NK-92 cells, HER2-CAR-NK cells and sPD-1-CAR-NK cells were placed in culture with cancer cell lines at different effector–target ratios for 6 h. The cytotoxicity of these three kinds of effector cells was measured by an LDH release assay. (**D**,**E**) NK-92 cells, HER2-CAR-NK cells and sPD-1-CAR-NK cells were incubated with JIMT-1 cells or MDA-MB-231 cells for 4 h. NK-92 cells which were not incubated with target cells were used as control group. The numbers of CD56+CD107a+ cells were detected by flow cytometry. (**F**) NK-92 cells, HER2-CAR-NK cells and sPD-1-CAR-NK cells were co-cultured with JIMT-1 cells (1 × 10^5^) for 12 h. The supernatants were collected, and an ELISA kit was used to detect the release of perforin (left), granzyme B (center) and IFN-γ (right). The data shown are representative of three experiments. Data represent the mean ± SD. n.s., nonsignificant; *** *p* < 0.001 by two-tailed Student’s *t*-test.

We then investigated the expression of HER2 and PD-L1 on the surface of different breast cancer cells, such as MDA-MB-231, MCF-7 and JIMT-1 cells. MDA-MB-231 cells are triple-negative human breast adenocarcinoma cells; MCF-7 cells are progesterone (PR)- and estrogen receptor (ER)-positive human breast epithelial adenocarcinoma cells [25]. The JIMT-1 cell line is derived from a patient with trastuzumab-resistant HER2-positive breast cancer [26]. As shown in Figure 2A,B, MDA-MB-231 cells were shown to have very low HER2 positivity with high PD-L1 expression, MCF-7 cells were shown to have high HER2 positivity with low PD-L1 expression and JIMT-1 cells were shown to have high HER2 positivity with high PD-L1 expression. We then compared the cytotoxicity of HER2-CAR-NK cells and sPD-1-CAR-NK cells against the three kinds of target cells, and NK-92 cells were used as the control group. Results are illustrated in Figure 2C. The lysis effects of HER2-CAR-NK cells on MDA-MB-231 cells were almost consistent with those of NK-92 cells, while HER2-CAR-NK cells showed stronger cytotoxicity to MCF-7 cells and JIMT-1 cells than NK-92 cells. Importantly, sPD-1-CAR-NK cells showed more potential killing capability towards these cancer cells than HER2-CAR-NK cells, most apparently to JIMT-1 cells, which expressed the highest levels of both HER2 and PD-L1. When the effector/target ratio was 10:1, the killing rate of JIMT-1 cells was more than 80% after treatment with sPD-1-CAR-NK cells, showing the excellent cytotoxic ability of sPD-1-CAR-NK cells. CD107a is a membrane marker of activated NK cells with degranulation. It was found that HER2-CAR-NK cells significantly induced the expression of CD107a compared with NK-92 cells, and sPD-1-CAR-NK cells further increased CD107a expression when co-cultured with target cells (Figure 2D,E). The cytolysis of NK cells has been well known to be associated with releases of granzyme B, perforin and IFN-γ. sPD-1-CAR-NK cells produced greater amounts of these functional molecules to deal with JIMT-1 cells than the HER2-CAR-NK cells detected by ELISA assay (Figure 2F). These results indicate that sPD-1-CAR-NK cells enhance cytotoxicity toward HER2-positive breast cancer cells and are more effective against HER2 and PD-L1 double-positive JIMT-1 cells.

### 2.3. sPD-1-CAR-NK Cells More Effectively Inhibit Growth of Target Humanized hHER2-EMT6 Xenografts

**Figure 3:**
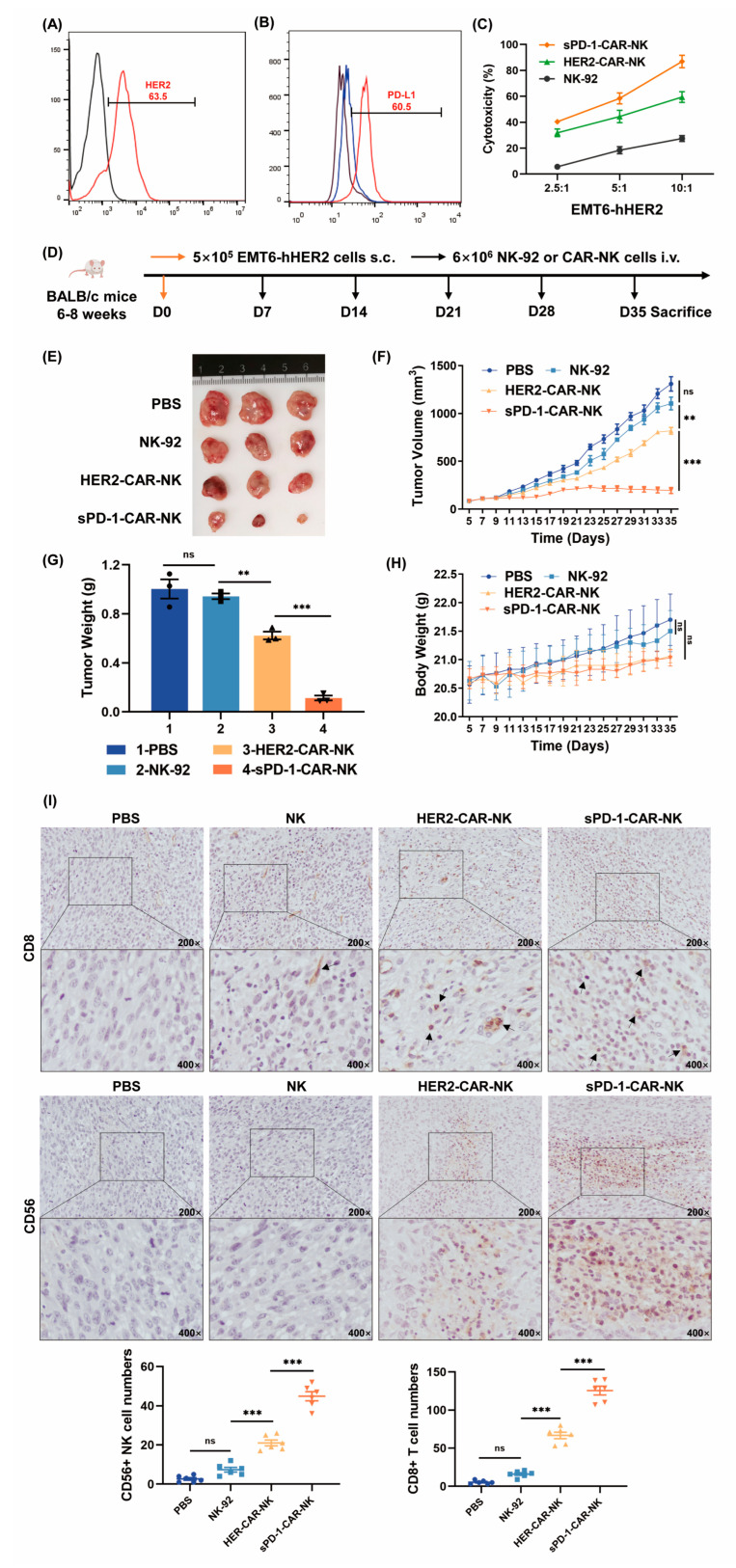
sPD-1-CAR-NK cells exhibit superior antitumor effects in immunocompetent BALB/c mice. (**A**,**B**) The expression of HER2 and PD-L1 on the surface of EMT6-hHER2 cells. A total of 5 × 10^6^ EMT6-hHER2 cells were stimulated with or without 50 ng/mL IFN-γ. The concentration was a proportional conversion based on the result of Figure 2F. (**C**) NK-92 cells, HER2-CAR-NK cells and sPD-1-CAR-NK cells were placed in culture with EMT6-hHER2 cells at different effector–target ratios for 6 h. The cytotoxicity of these three kinds of effector cells was measured by an LDH release assay. (**D**) Experimental timeline. BALB/c mice were injected with 5 × 10^5^ EMT6-hHER2 cells (i.v.); from the seventh day, 6 × 10^6^ NK-92 cells, HER2-CAR-NK cells or sPD-1-CAR-NK cells were injected into the tail vein every seven days. After 34 days, the mice were euthanized. (**E**,**F**) Photographs of tumors and statistical chart of tumor volume. (**G**,**H**) Statistical chart of tumor weight and mouse body weight. (**I**) The summarized data and representative results of CD8 and CD56 staining of tumors by immunohistochemical staining analysis. Data shown represents at least three independent experiments. n.s., nonsignificant; ** *p* < 0.01; *** *p* < 0.001 by two-tailed Student’s *t*-test or two-way ANOVA with Dunnett’s test.

After the functional verification of sPD-1-CAR-NK cells in vitro, we firstly selected a conventional animal model to explore the antitumor effect of sPD-1-CAR-NK cells in immunocompetent BALB/c mice. An EMT6 cell line was established from a transplantable murine mammary carcinoma that arose in BALB/c mice. We modified EMT6 cells to highly express human HER2 protein for an in vivo study, and the expression of PD-L1 and HER2 are shown in Figure 3A,B. In Figure 3B, EMT6 cells presented lower expressed PD-L1; however, it was upregulated prominently after IFN-γ treatment. It was found that sPD-1-CAR-NK cells more markedly facilitated lysis toward EMT6-hHER2 cells than HER2-CAR-NK cells (Figure 3C). EMT6-hHER2 cells were then administered subcutaneously into BALB/c mice. When the tumor volume reached 100 mm^3^ (day 7), mice bearing the tumor were randomly allocated into four groups (*n* = 3) and assigned to receive one of the following intravenous injections: (1) sterile PBS, (2) 6 × 10^6^ NK-92 cells, (3) 6 × 10^6^ HER2-CAR-NK cells or (4) 6 × 10^6^ sPD-1-CAR-NK cells. Treatments were administered every 7 days until the end of the study. The results showed that HER2-CAR-NK cells evidently inhibited tumor growth with a greater reduction in tumor volume compared to NK-92 cells, and sPD-1-CAR-NK cells exhibited a more dramatic delay in tumor growth than HER2-CAR-NK cells (Figure 3E,F). Similar results were obtained in tumor weight (Figure 3G). Mice in all groups injected with various NK cells did not exhibit any significant changes in body weight compared with the PBS control (Figure 3H). The infiltrations of NK and T cells into tumor tissue were also investigated by immunohistochemical staining analysis. The numbers of NK and T cells were increased in HER2-CAR-NK cells and sPD-1-CAR-NK cells groups compared with those in the NK-92 group, and were two-fold greater in the sPD-1-CAR-NK cell group than in the HER2-CAR-NK group (Figure 3I). These data indicate that sPD-1-CAR-NK cells promote antitumor efficiency by improving the tumor microenvironment.

### 2.4. Co-Expression of sPD-1 with HER2-CAR in NK-92 Cells Has Antitumor Efficacy Superior to HER2-CAR Plus sPD-1

**Figure 4:**
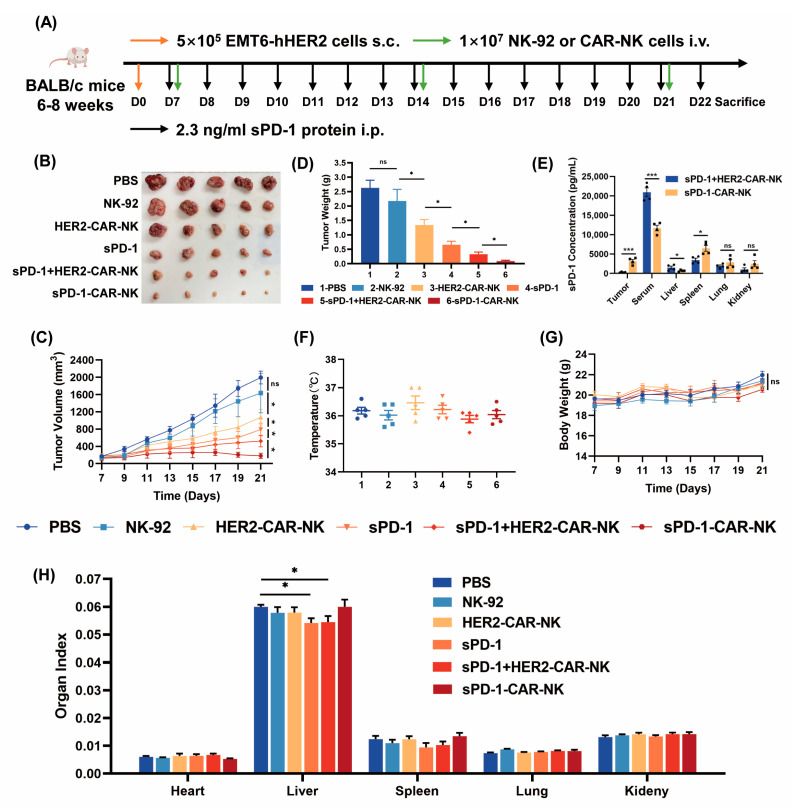
The antitumor effects of sPD-1-CAR-NK cells have more potential than those of HER2-CAR-NK cells combined with sPD-1 protein. (**A**) Experimental timeline. BALB/c mice were injected with 5 × 10^5^ EMT6-hHER2 cells (i.v.). From the seventh day, 1 × 10^7^ NK-92 cells, HER2-CAR-NK cells or sPD-1-CAR-NK cells were injected into the tail vein every 7 days. sPD-1 protein was administered daily in mice in the sPD-1 group (i.p.), with a concentration of approximately 2.4 ng/mL. The combination group was administrated sPD-1 protein daily and HER2-CAR-NK cells every week. The experiment ended on the 22nd day, and the mice were euthanized. (**B**,**C**) Tumor photographs and statistical chart of tumor volume. (**D**) Statistical chart of tumor weight. (**E**) An ELISA kit was used to detect the sPD-1 content in serum and homogenates of various organs. (**F**) The body temperature of the mice was measured at the end of the experiment. (**G**) Statistical chart of mice body weight. (**H**) The organ indices of mice. Data shown represents at least three independent experiments. n.s., nonsignificant; * *p* < 0.05, *** *p* < 0.001 by two-tailed Student’s *t*-test or two-way ANOVA with Dunnett’s test.

In order to verify the results, it was necessary to engineer the extracellular domain of PD-1 into the HER2-CAR gene. We performed animal experiments to compare the antitumor effect of sPD-1-CAR-NK cells with that of HER2-CAR-NK in combination with the sPD-1 protein. EMT6-hHER2 cells were administered subcutaneously to BALB/c mice aged 6–8 weeks. The experimental scheme is shown in Figure 4A. A week later, when tumor volumes reached 100 mm^3^, the mice were randomized into six groups. The sPD-1 protein was intraperitoneally injected daily into animals, and 6 × 10^6^ NK-92 cells, HER2-CAR-NK cells or sPD-1-CAR-NK cells were injected intravenously (i.v.) every seven days. The combination group was administered the sPD-1 protein daily and HER2-CAR-NK cells every seven days. sPD-1 was injected at a concentration of 2.4 ng/mL, which was equivalent to the amount of sPD-1 in the culture supernatant of 6 × 10^6^ sPD-1-CAR-NK cells. The experiment ended on the 22nd day. It was discovered that the antitumor effect of sPD-1-CAR-NK cells was much stronger than that of the HER2-CAR-NK cells, as before. Notably, the tumor size in mice treated with sPD-1-CAR-NK cells significantly decreased compared to that in mice with HER2-CAR-NK cells in combination with the sPD-1 protein, and similar results were obtained with tumor weight (Figure 4B–D). The contents of sPD-1 in the serum and various tissues were detected by an ELISA assay. As shown in Figure 4E, the combination of HER2-CAR-NK cells and sPD-1 protein had higher levels of sPD-1 in the serum than the sPD-1-CAR-NK cells, as well as much lower amounts of sPD-1 in the tumor tissues than the sPD-1-CAR-NK cells. It was also noted that the sPD-1 levels in the spleen of the sPD-1-CAR-NK cell group were also above those in the combination-treated mice, and evidently lower in the live tissue. There was no obvious difference in liver, lung and kidney tissue in the two groups. CAR-T cells are subjected to a cytokine storm leading to treatment failure. A fever phenomenon often occurs alongside a cytokine storm in mice [27]. One advantage of CAR-NK over CAR-T is that it is not easy to produce inflammatory storms [28]; thus, we measured the body temperatures of mice before the termination of the experiment. The results showed that the body temperature of the mice in each group were not altered compared to the PBS group (Figure 4F). Moreover, there was no significant change in mouse weight for all groups (Figure 4G). At the end of the experiment, several organs were blunt dissected and organ indices were calculated. It was found that organ indexes did not decline in the all groups compared with the PBS group, except for a significant decrease in liver index in the sPD-1 group and the combination group (Figure 4H). Altogether, these results indicate that adding sPD-1 into the HER2-CAR gene expressed in NK-92 cells has antitumor efficacy superior to that of HER2-CAR-NK cells plus sPD-1 in vivo, and sPD-1-CAR-NK cells are as safe as HER2-CAR-NK cells in anticancer applications.

### 2.5. sPD-1-CAR-NK Cells More Effectively Block the Growth of JIMT-1 Cell Xenografts in Immune Humanized NOG Mice

**Figure 5:**
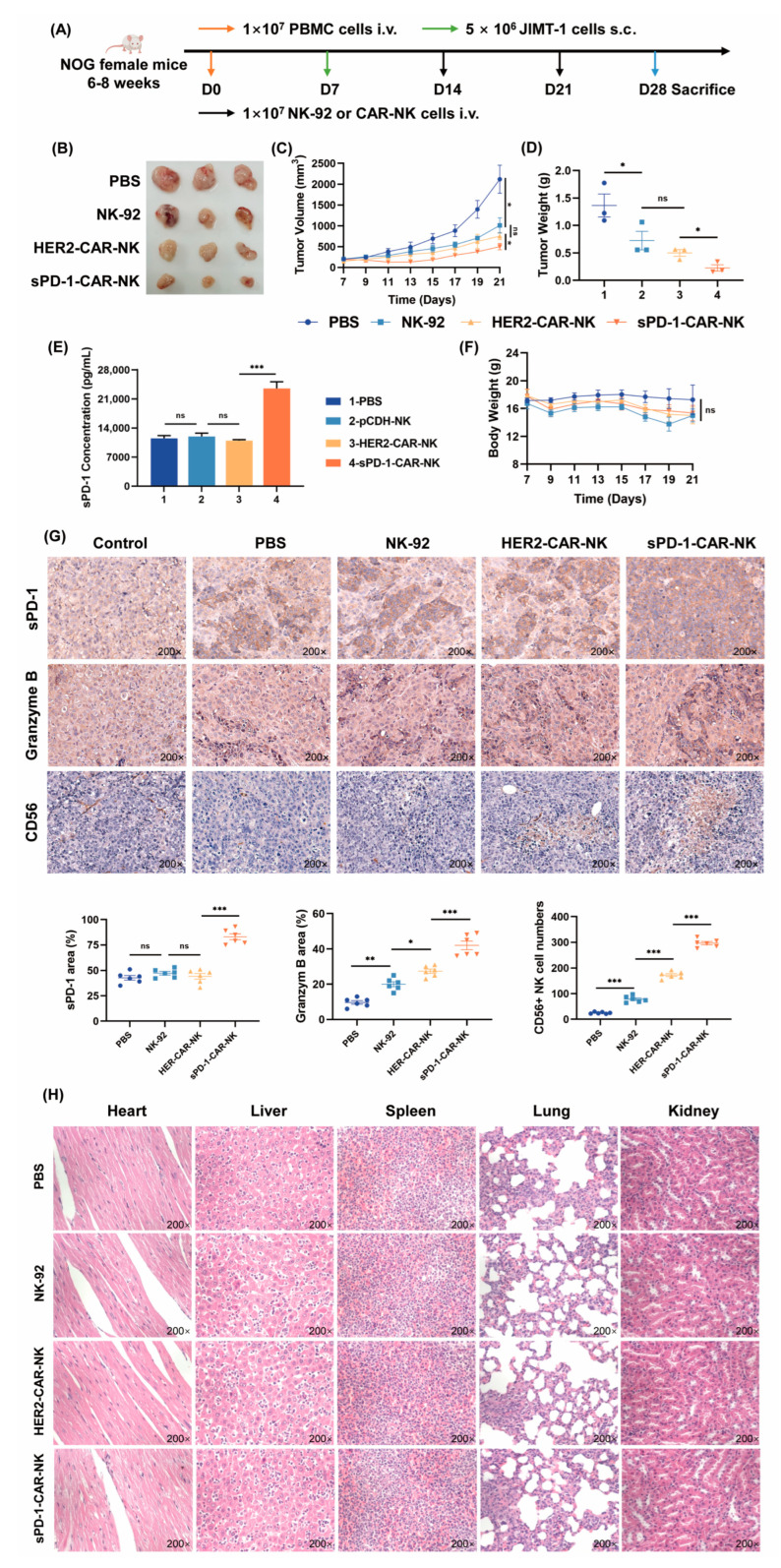
sPD-1-CAR-NK cells show significant antitumor effects in immune humanized NOG mice. (**A**) Experimental timeline. NOG mice were injected with 1 × 10^7^ PBMC (i.v.), seven days later (day 7), 5 × 10^6^ JIMT-1 cells were injected subcutaneously. Then, 1 × 10^7^ NK-92 cells, HER2-CAR-NK cells or sPD-1-CAR-NK cells were injected into the tail vein every 7 days. (**B**,**C**) Tumor photographs and statistical chart of tumor volume. (**D**) Statistical chart of tumor weight. (**E**) At the end of the experiment, an ELISA kit was used to detect the sPD-1 in the serum of different groups of NOG mice. (**F**) Statistical chart of mouse body weight. (**G**) The summarized data and representative results of sPD-1, granzyme B and CD56 staining of tumors by immunohistochemical staining analysis. Control tissues were incubated with the antibody diluent alone, followed by incubation with secondary antibodies and detection reagents. (**H**) Histological images of hematoxylin–eosin (H&E) staining. All images were obtained at 200× magnification. Data shown represents at least three independent experiments. n.s., nonsignificant; * *p* < 0.05, ** *p* < 0.01, *** *p* < 0.001 by two-tailed Student’s *t*-test or two-way ANOVA with Dunnett’s test.

We lastly evaluated the efficacy of sPD-1-CAR-NK on the growth of tumors with resistance to trastuzumab therapy in the presence of human immunologic cells. Remarkably, immunodeficient NOG mice are readily grafted by human PBMC and human JIMT-1 tumor cells. As illustrated in Figure 5A, the NOG mice were reconstructed by intravenous injection (i.v.) with a healthy human donor PBMC (1 × 10^7^ cells). After seven days, the JIMT-1 cells were inoculated s.c. into the right back flank of the mice with 5 × 10^6^ cells. When tumors reached 200 mm^3^ (day 14), NK-92 cells, HER2-CAR-NK cells or sPD-1-CAR-NK cells were administered, i.v. separately once a week for 2 weeks. The results showed that sPD-1-CAR-NK cells still displayed the best inhibition capacity against JIMT-1 xenografts in immune humanized NOG mice. We noted that compared to PBS, NK-92 and HER2-CAR-NK cells showed significant inhibitory effects, and sPD-1-CAR-NK cells presented more inhibition ability against tumors compared to HER2-CAR-NK cells (Figure 5B–D). We also detected the levels of sPD-1 in the serum of immune humanized mice after treatment with sPD-1-CAR-NK cells for 24 h and found that sPD-1-CAR-NK cells greatly increased the amount of sPD-1 (Figure 5E). In tumor tissues, the protein content of sPD-1 and the infiltration of NK cells and their activation were detected by immunohistochemical staining analysis. As shown in Figure 5G, sPD-1 was heavily distributed in the tumor tissues of the group treated with sPD-1-CAR-NK cells. Both HER2-CAR-NK cells and sPD-1-CAR-NK cells promoted NK cell infiltration into the tumor tissue and increased release of granzyme B, and sPD-1-CAR-NK cells displayed more potential than HER2-CAR-NK cells. There was no significant difference in body weight between the constructed-NK-cell-treated mice and the PBS control mice (Figure 5F). To discover if the sPD-1-CAR-NK cells impaired important organs in mice, an H&E staining assay was carried out. Results showed that, similar to the PBS group, no significant changes were found in the heart, liver, spleen, lung and kidney tissue of mice after treatment with various NK cells (Figure 5H). All these results support the idea that sPD-1-CAR-NK can more effectively activate cancer immunity antagonist growth of trastuzumab-resistant breast cancer cells in vivo.

## 3. Discussion

The overexpression of the HER2 protein largely contributes to tumor progression and metastasis, and several studies have investigated the therapeutic potential of HER2-CAR-NK cell-based therapies in breast tumors, which induce the selective elimination of tumor cells in orthotopic breast carcinoma xenografts [19,29]. Despite these advances, research on the clinical development of CAR-engineered NK cells is still limited. NK cell expansion ex vivo also promotes the expression of some immune checkpoint receptors such as PD-1, thereby decreasing CAR-NK therapy efficacy [30]. IFN-γ produced by activated NK cells also augments PD-L1 expression in cancer cells [7]. In addition, trogocytosis has been described recently as a new mechanism whereby NK cells acquire PD-1 from tumor cells [31]. Therefore, blocking the interaction of PD-1–PD-L1 is an alternative way to improve the anticancer efficacy of HER2-CAR-NK cells. 

In addition to full-length PD-1, four splice variants of PD-1 mRNA have been cloned from human peripheral blood mononuclear cells (PBMCs) [32]. The sPD-1 in our study, constructed into HER2-CAR or complementing it, was a splice variant, PD-1Deltaex2, which lacks exon 2 and encodes the extracellular domain [33]. Due to the properties of sPD-1, it may break immunosuppression by blocking its ligand. In fact, patients with high sPD-1 levels had longer survival than those with low sPD-1 levels [34], and sPD-1 has fewer side effects than monoclonal antibodies, while monoclonal antibodies exert strong therapeutic effects [35]. Given that parental NK-92 cells provide unlimited homogeneous effectors with easy manufacturing expansion and genetic manipulation [36], we generated stable clonal NK-92 cells that express humanized sPD-1-CAR based on HER2-specific antibody-harboring CD28- and CD3ζ-signaling domains. Our data demonstrated that sPD-1-CAR-NK cells specifically and efficiently killed the cancer cells with high expression of HER2, indicating that the incorporation of sPD-1 significantly fosters the antitumor activity of HER2-CAR-NK cells.

Trastuzumab binds to a membrane proximal epitope in the extracellular region of HER2. Although trastuzumab constitutes a breakthrough in the treatment of advanced breast cancer, 70% of HER2-overexpressing breast cancers show resistance to trastuzumab as a single agent [37]. JIMT-1 cells are a line established from a trastuzumab-resistant breast cancer patient [38]. Seyedmirzaei and his coworkers found that a single dose of HER2-specific CAR-T cells eliminated tumors and improved long-term survival in trastuzumab-resistant breast-tumor-cell-bearing mice [39]. It appears that CAR-T cells could penetrate the tumor matrix, which is usually referred to as a barrier for antibody drugs [20]. In addition, HER2-specific CAR-T can stimulate more cytotoxicity than trastuzumab. Our studies demonstrate that HER2-CAR-NK cells significantly inhibit JIMT-1 cell xenografts, and sPD-1 engineered into HER2-specific CAR further enhanced the inhibitory effect against trastuzumab-resistant breast tumors.

PD-1, a type I transmembrane receptor, is the most extensive inhibitory receptor expressed in various immune cells and activated vascular endothelial cells [40], and sPD-1 can interfere with the PD-1 in these immune cells’ interaction with their ligand [10]. To reflect the multiple immune effects of sPD-1 secreted by sPD-1-CAR-NK cells in vivo, we performed animal experiments in the target humanized immunocompetent mice and immune system humanized mice. The humanized mouse model of the immune system has both human immune system and human tumor cells, which can better simulate the human tumor immune microenvironment and show the interaction between the two [41]. Therefore, we also constructed a humanized immune system mouse model to verify the antitumor effect of sPD-1-CAR-NK cells on JIMT-1 human breast cancer xenografts. In these models, sPD-1-CAR-NK cells had more beneficial anticancer effects than HER2-CAR-NK cells. Furthermore, we demonstrated that sPD-1-CAR-NK cells exhibited more potential antitumor efficacy than HER2-CAR-NK cells plus sPD-1, because there was more sPD-1 in the tumor tissue in the mice treated with sPD-1-CAR-NK cells (Figure 5E). In addition, the level of sPD-1 was higher in serum and lower in tumor in the combination group than in the sPD-1-CAR-NK cell group (Figure 4E). These results indicate that sPD-1 was enriched in the tumor microenvironment due to the sPD-1-CAR-NK cells targeting HER2. It was noted that there was a high level of sPD-1 in the serum up to nanomolar concentration, indicating a high expression efficiency of the vector with the sPD-1-CAR gene. Activated NK cells typically result in the enhancement of endogenous antitumor immune responses in an immunocompetent host [42]. Consistently, more NK cells, T cells and activated cytokine granzyme B were enhanced after treatment with sPD-1-CAR-NK cells in vivo.

CAR-NK immunotherapy emerges as a safer, faster and more cost-effective approach without the severe toxicities associated with CAR-T cells, such as on-target off-tumor toxicities, cytokine release syndrome (CRS) or immune-effector-cell-associated neurotoxicity syndrome (ICANS) [43]. In line with these reports, we found no significant changes in the mouse weight and body temperature, and no apparent organ injury in the heart, live, spleen, lung or kidney in the mice treated with the engineered CAR-NK cells. There were decreases in live coefficients in the group with sPD-1 injection alone and the group with combination treatment, which was consistent with the report that liver immune-related adverse events are frequently observed in cancer patients treated with immune checkpoint inhibitors due to necrotic hepatocytes [44]. These results indicate that sPD-1 established into HER2-CAR-NK cells has good safety in vivo.

## 4. Materials and Methods

### 4.1. Cell Lines and Cell Culture Conditions

All the cells were obtained from the Cell Bank of the Chinese Academy of Sciences (Shanghai, China) and cultured at 37 °C with 5% CO_2_ under fully humidified conditions with the above media (HyClone, Logan, UT, USA), supplemented with 10% fetal bovine serum FBS (Gibco, Grand Island, NY, USA), 100 U/mL penicillin (Solarbio, Beijing, China) and 100 μg/mL streptomycin (Solarbio, Beijing, China). NK-92 cells were cultured in Alpha Minimum Essential Medium (HyClone, Logan, UT, USA), supplemented with 12.5% horse serum (Solarbio, Beijing, China), 12.5% fetal bovine serum (FBS, HyClone, Logan, UT, USA) and 100–200 U/mL recombinant IL-2 (PeproTech, Cranbury, NJ, USA). MDA-MB-231 cells were cultured in Leibovitz’s L-15 medium (HyClone, Logan, UT, USA) with 15% fetal bovine serum (FBS, HyClone, Logan, UT, USA). MCF-7, EMT6 and EMT6-hHER2 cells were cultured in Roswell Park Memorial Institute (RPMI)-1640 medium (HyClone, Logan, UT, USA), and 293T, NIH3T3, NIH3T3-hHER2 and JIMT-1 cells were cultured in Dulbecco’s modified Eagle’s medium (DMEM, HyClone, Logan, UT, USA). NIH3T3-hHER2 cells were constructed and conserved by our laboratory. EMT6-hHER2 cells were kindly donated by the laboratory of Meiyu Geng, Shanghai Institute of Pharmaceutical Research.

### 4.2. Isolation and Purification of PBMC

Human blood samples from healthy adult donors were obtained from the Qilu Hospital of Shandong University (Qingdao) under protocols (KYLL-2016038) approved by the ethics committee. The working solution was prepared according to the instructions of the human peripheral blood mononuclear cell isolation kit (Solarbio, Beijing, China). Fresh anticoagulated whole blood was collected and mixed with pH 7.2 phosphate buffer (PBS) at a 1:1 ratio. The working solution was carefully layered with the same volume of diluted blood sample, avoiding any mixture. The samples were centrifuged at 1200× *g* for 30 min at room temperature. At the end of the centrifugation, the liquid in the tube was divided into three layers, with PBMCs in the middle layer. The intermediate layer was transferred to a new centrifuge tube and washed three times with PBS.

### 4.3. Construction of Lentiviral CAR-Expression Vector

HER2-CAR comprised a single-chain variable fragment targeting HER2, the hinge domain of the CD8α molecule, the transmembrane region and the intracellular signaling domain of the human CD28 molecule, and the intracellular signaling domain of the CD3ζ molecule. sPD-1-CAR comprised PD-1 extracellular domain (ECD, AA1-155), 2A self-cleaving peptides (F2A), a single-chain variable fragment targeting HER2, the hinge domain of the CD8α molecule, the transmembrane region and the intracellular signaling domain of the human CD28 molecule, and the intracellular signaling domain of CD3ζ molecule. CAR expression is driven by the CMV promoter and EF1α promoter. Cop Green fluorescent protein was used as a reporter gene.

Specific primers were designed for PCR amplification of above coding genes, and the amplified products were sequentially linked. Chimeric antigen receptor HER2-CAR and enhanced sPD-1-CAR coding gene were obtained.

The chimeric antigen receptor HER2-CAR and sPD-1-CAR coding genes were cloned into the lentiviral vector pCDH-CMV-MCS-EF1-copGFP (the vector was obtained from Addgene) to obtain the recombinant lentiviral vector. 

### 4.4. Lentivirus Production

The bacterial liquid of pSPAX2 plasmids, PMD2G plasmids and the lentiviral-vector-containing CAR structure were cultured overnight on LB solid medium containing 50 μg/mL ampicillin resistance. The next day, monoclonal colonies were transferred to LB liquid medium (5 mL) containing ampicillin (50 μg/mL) for further culturing. The plasmids were extracted according to the instructions of the plasmid mini-extraction kit (Omega Bio-Tek, USA), and their concentrations were determined using an Epoch2 enzyme marker. 

A total of 5 × 10^6^ 293T cells were cultured in a 10 cm petri dish. When 60–70% of the petri dish was covered with cells, the Lipo3000 method was used for transfection. After 72 h of transfection, the virus was harvested from the conditioned medium and filtered using a 0.45 µm filter unit (Millipore, United States) to remove cell debris. 

### 4.5. Generation of HER2-CAR-NK Cells and sPD-1-CAR-NK Cells

NK-92 cells at 2 × 10^5^ cells/well were inoculated in 24-well plates, and HER2-CAR and sPD-1-CAR viral solutions (2, 5, 8, 10 μL/well) were added to each well with 8 μg/mL of polybrene. Cells were cultured in an incubator at 37 °C for 10 h. The virus supernatant was removed by centrifugation and replaced with fresh medium. The cell lines stably expressing the specific gene were obtained by selecting them in medium containing 8 μg/mL puromycin (Sigma-Aldrich, St. Louis, Missouri, USA).

### 4.6. Flow Cytometry Analysis

The CAR frame was coupled with the tagged protein GFP, so the surface of the successfully transfected NK-92 cells emitted green fluorescence. NK-92 cells and CAR-NK cells were collected by centrifugation and washed with PBS (containing 1% FBS) three times. The fluorescence intensity was detected by flow cytometry (Calibur, BD Biosciences, Franklin Lakes, NJ, USA). 

MDA-MB-231, MCF-7, JIMT-1, NIH3T3-hHER2 and EMT6-hHER2 cells were digested by 0.25% trypsin EDTA, resuspended in 100 µL of FACS buffer (1% BSA in PBS) and then incubated for 1 h with allophycocyanin (APC)-conjugated anti-PD-L1 antibodies or allophycocyanin (APC)-conjugated anti-HER2 antibodies. After undergoing washing, the cells were incubated for 30 min in the dark with an Alexa Fluor 488-conjugated secondary antibody. After further washing, the cells were run through a flow cytometer (Calibur, BD Biosciences, Franklin Lakes, NJ, USA). 

NK-92 cells or CAR-NK cells were co-cultured with target cells at the effector–target ratio of 10:1 for 4 h. The cells were collected by centrifugation and stained with anti-human CD56 and anti-human CD107a antibodies for 30 min after washing. After further cleaning, the cells were collected by flow cytometry (Calibur, BD Biosciences, Franklin Lakes, NJ, USA).

The results of the flow cytometry are presented as percentages of the positive fluorescent cells. FlowJo software (BD Biosciences, Franklin Lakes, NJ, USA) was used for data analysis.

### 4.7. Analysis of Cytokine and sPD-1 Protein Levels by ELISA

After NK-92 cells, HER2-CAR-NK cells and sPD-1-CAR-NK cells were cultured for 24 h, the supernatant was collected by centrifugation, and the content of sPD-1 protein in the supernatant was detected according to the instructions of the Human PD-1/PDCD1 ELISA Kit (Sino Biological, Beijing, China).

Blood was taken from mice, left for 2 h at room temperature, and centrifuged at 10,000× *g* to extract the serum. The organs of the mice were taken and cut into pieces, and five times the volume of PBS was added, and they were homogenized and centrifuged at 10,000× *g* to extract the supernatant. The contents of the sPD-1 protein in the serum or supernatant were detected according to the instructions of the Human PD-1/PDCD1 ELISA Kit (Sino Biological, Beijing, China).

Enzyme-linked immunosorbent assay (ELISA) kits for human IFN-γ, granzyme B and perforin were purchased from Dakewe (Beijing, China), and all ELISA assays were performed according to the manufacturer’s protocols. NK-92 cells, HER2-CAR-NK cells and sPD-1-CAR-NK cells were co-cultured with JIMT-1 cells at a 5:1 E/T ratio for 12 h, and then the culture supernatants were collected and analyzed by ELISA kits.

### 4.8. Western Blot Analysis

Harvested cells were homogenized in RIPA supplemented with protease inhibitor cocktail at 4 °C for 30 min. Equal amounts of protein per sample were fractionated by SDS-PAGE. The protein bands were transferred onto nitrocellulose filter membranes. The membranes were blotted with primary antibodies followed by secondary antibodies. Finally, the membranes were imaged by enhanced chemiluminescence. 

### 4.9. Cytotoxicity Assay

NK-92 cells, HER2-CAR-NK cells and sPD-1-CAR-NK cells were placed in culture with different cancer cell lines at different effector–target ratios (2.5:1, 5:1 or 10:1) for 6 h. The cytotoxicity of these three kinds of effector cells was measured by a lactate dehydrogenase (LDH) assay kit (Beyotime Institute of Biotechnology, Shanghai, China). 

### 4.10. Mouse Xenograft Studies

BALB/c mice were purchased from the Jinan Pengyue Experimental Animal Center. NOG (NOD.CB17-PrkdcscidIl2rgtm1Il6tm1(IL6)/Bcgen) mice were purchased from the Charles River Laboratories Animal Technology Company Limited. All animal experiments were performed by the guidelines and regulations approved by the Committee on the Ethics of Animal Experiments of Ocean University of China. All mice were housed in specific pathogen-free conditions.

BALB/c mice were injected subcutaneously with EMT6-hHER2 cells. When the tumor volume reached 100 mm^3^, mice bearing the tumors were randomly allocated into groups. Tumor growth was measured by caliper measurements, and tumor volume was calculated using the following formula: V = 1/2 × a × b × b, where V = tumor volume, a = maximum tumor diameter and b = minimum tumor diameter. The mice were treated with different immune cells injected weekly through the tail vein, while IL-2 was injected intraperitoneally to maintain the activity of immune cells. The sPD-1 treatment group and combined treatment group were intraperitoneally injected with sPD-1 protein solution (2.4 ng/mL) every day. When the mean tumor volume in the control group reached 1500–2000 mm^3^, mice were euthanized by cervical dislocation under carbon dioxide inhalation.

A total of 1 × 10^7^ PBMCs were injected into NOG mice through the caudal vein once a week to construct humanized mice with a human immune system. After successful construction, mice were inoculated with 5 × 10^6^ JIMT-1 cells subcutaneously. When the tumor volume reached 100 mm^3^, mice bearing the tumor were randomly allocated into groups. The mice were treated with different immune cells injected weekly through the tail vein, while IL-2 was injected intraperitoneally to maintain the activity of immune cells. When the mean tumor volume in the control group reached 1500–2000 mm^3^, mice were euthanized by cervical dislocation under carbon dioxide inhalation.

### 4.11. Immunohistochemistry

SP-9001 SPlink Detection Kits (Biotin-Streptavidin HRP Detection Systems) and SP-9002 SPlink Detection Kits (Biotin-Streptavidin HRP Detection Systems) were used for conventional immunohistochemistry (IHC). Tumor tissues were fixed in 4% paraformaldehyde; the paraffin-embedded tissue sections (4 µm) were deparaffinized in xylene, rehydrated in graded alcohol and placed in 90℃ sodium citrate buffer for 10 min. Then, they were treated with 3% hydrogen peroxide to block endogenous peroxidase. Nonspecific sites were blocked with animal nonimmune serum (Maxvision) and incubated with diluted rabbit or mouse anti-human primary antibodies overnight at 4 °C. After incubation with the corresponding secondary antibody for 15 min at room temperature, the sections were stained using DAB color development solution. Cell nuclei were stained with hematoxylin (Sigma). The images were acquired at 200× magnification using a ZEISS Axioskop2 plus advanced positive microscope. 

### 4.12. Statistical Analysis

The data were analyzed using GraphPad Prism 5.0 (GraphPad Inc., San Diego, CA, USA) software. All data are expressed as the mean ± SD. Statistical analyses were conducted by two-tailed Student’s *t*-test or one-way ANOVA. Two-way ANOVA with multiple comparisons was used to compare tumor growth over time. Dunnett’s test was performed for multiple comparison post-tests. n.s., nonsignificant; * *p* < 0.05, ** *p* < 0.01 and *** *p* < 0.001 were considered statistically significant.

## 5. Conclusions

In summary, we have generated new chimeric antigen receptor NK cells by recruiting sPD-1 into HER2 and PD-L1 highly expressed breast cancer cells to improve the anticancer efficacy of HER2-CAR-NK in HER2-positive breast tumors, including trastuzumab-resistant breast cancer tumors. Our findings demonstrated that sPD-1- CAR-NK cells are promising immunoediting cells worthy of further development for clinical application.

## Data Availability

The data presented in this study are available on request from the corresponding authors.
